# Further Notes on Compulsory Notification

**Published:** 1907-12-21

**Authors:** 


### Further Notes on Compulsory Notification.

The interest which the profession is taking in the Early Notification of Births Act renders unnecessary any apology for a further reference to the subject. In previous articles (*see* The Hospital, July 6th and November 9th) we have defined our own attitude to the question, and to judge from various public reports, not to speak of many private communications which have reached us, the view we have advocated is, in a steadily increasing degree, receiving the support of our fellow practitioners. We continue to urge that every effort shall be made to persuade local authorities not to adopt the Act. The Act is distinctly unjust to the profession in respect to the fact that it claims public service from the individual without any recognition in the shape of a fee. But, of still more importance, as we think, it is objectionable inasmuch as it places private medical practitioners in a position where they are compelled to reveal to the public authorities information regarding the affairs of individual patients. We are not contending against the claim that the State has a right to demand the notification of every birth within 36 hours of its occurrence. What we object to is the proposal to use private medical practitioners as the agents for the collection of this information. And our objection is founded on the proposition that such a proceeding tends to weaken the faith of the public in the confidential character of communications made by patients to their medical advisers, and this faith we hold to be a substantial public interest.

Upon the view thus stated one or two criticisms have been passed, and to these it is appropriate that allusion should here be made. It has, for example, been argued that as the medical practitioner, in notifying a birth in accordance with the provisions of the new Act, will be acting under the direction of the law, there can be no charge of breach of confidence. Doubtless in the technical sense this is correct—that is to say, the practitioner cannot for such action be sued for damages in a court of law. But the question must be considered in a larger spirit than that of mere legal obligation. It is necessary to ask whether the general moral sense of the community will approve the revelation by the private medical adviser of the patient of information which in a certain number of instances will cause public gossip and scandal, and also pain and shame to the individuals concerned. Once more, we repeat, this is not to argue that the information in question ought not to be given to the public authorities. But it is a claim that the instrument of such a purpose should not be the patient's confidential medical adviser. There are more instances than one in which what is legally defensible fails to carry the general justification of the community. Thus there is no legal obligation to pay money lost in gambling or betting; but the individual who avails himself of this fact in order to avoid the payment of a "debt of honour" is hardly considered an honest man. Equally we can conceive circumstances in which a medical practitioner, though able to plead legal compulsion, may yet find himself the subject of public opprobrium. It must be remembered that large numbers of the public trust their most secret affairs to members of the medical profession, and what may seem a breach of faith under one particular heading is highly likely to lead to doubt and uncertainty in other directions. Alter the relations between the patient and his medical adviser in one respect, and you can hardly avoid modifying, at least in some degree, the entire position. Hence, while the notification of a birth may not be a breach of confidence in law, it certainly involves for the practitioner a public declaration which does not come within the scope of his duties as the patient's confidential adviser, and it is in this capacity, it must be remembered, that he is consulted, and for these duties he is paid a fee.

Again, it has been urged that early notification of births is a large public interest, and that medical practitioners are in the best position to promote this. But the confidence of the public in the secrecy of the consulting-room is also a large public interest, and the adoption of the new Act, as we have tried to show, will certainly weaken this. It must be judged which of the two claims is the more important. The course of justice is another large public interest, and were legal advisers compelled in the witness-box to reveal the communications they had received from their clients, this interest would often be promoted. But sooner or later such a proceeding would be followed by a general loss of confidence in the honour and secrecy of solicitors, and such a result, it is recognised, would be prejudicial to the community. To gain an immediate and individual advantage may mean ultimately a large public loss, and this is, in our judgment, what is likely to occur if medical practitioners are forced to appear as betrayers of the confidence of their patients.

